# Zebrafish Embryo Toxicity of a Binary Mixture of Pyrethroid Insecticides: d-Tetramethrin and Cyphenothrin

**DOI:** 10.1155/2018/4182694

**Published:** 2018-12-26

**Authors:** Janthri C. Mendis, Thejani K. Tennakoon, Chanika D. Jayasinghe

**Affiliations:** Department of Zoology, Faculty of Natural Sciences, The Open University of Sri Lanka, Nawala, Nugegoda, Sri Lanka

## Abstract

Pesguard FG161™, a mixture of d-tetramethrin and cyphenothrin (1:3 ratio), is extensively used to achieve rapid control of adult dengue vector,* Aedes aegypti, *during the disease outbreaks. Both d-tetramethrin and cyphenothrin are synthetic pyrethroids that are known to have adverse effects on non-mammalian organisms such as fish. The present study intended to use zebrafish embryo toxicity model to investigate the toxic effect of the above binary mixture on fish. Particularly, zebrafish embryo toxicity model provides an alternative to acute fish toxicity tests in terms of animal welfare perspective as the embryos are not considered live until 5 days after fertilization. The zebrafish embryos (2 hrs after fertilization) were exposed to a binary mixture of pyrethroids at different concentrations (d-tetramethrin: 0.01 – 1.20 *μ*molL^−1^ and cyphenothrin: 0.03 – 3.20 *μ*molL^−1^) for 24, 48, and 72 hrs at room temperature (26°C) according to the OECD guideline no. 236. Percentage mortality of embryos were calculated by observing the lethal endpoints and LC_50_ values were calculated for each time interval employing the probit analysis. This binary mixture was highly toxic to zebrafish embryos and was found to be concentration and time dependent. LC_50_ values at 24 hrs (d-tet: 0.58 *μ*molL^−1^, cyp: 1.74 *μ*molL^−1^) were significantly reduced in 48 hrs (d-tet: 0.11 *μ*molL^−1^, cyp: 0.33 *μ*molL^−1^) and 72 hrs (d-tet: 0.03 *μ*molL^−1^, cyp: 0.09 *μ*molL^−1^). Coagulation of embryos was the most common lethal effect observed and lack of somite formation and lack of heartbeat were also observed. The present study revealed that the binary mixture is highly toxic to zebrafish embryos even when based on nominal concentrations. Hence, extensive use of these pesticides could be detrimental to fish population and integrated vector control methods which involve the minimum use of insecticides are recommended. Further, this study highlights the applicability of zebrafish embryo toxicity model as an alternative method to investigate the toxicity of pyrethroids to fish.

## 1. Introduction

Dengue is a major mosquito-borne viral disease that affects more than 125 countries. The global incidence of dengue has reached epidemic levels with a 30-fold increase in the last five decades [1]. Dengue causes around 500 million cases every year with 1 and 3 million deaths. Also, the economic and social burden of dengue is enormous [2].

Despite the advances in the development of vaccines and chemotherapy, mosquito vector control remains the most effective and principal strategy of dengue control [3]. Vector control efforts have been successfully used for decades to combat dengue infection. Current vector control strategies include combinations of chemical and biological agents that target different stages of mosquitoes and management of breeding sites [1]. Dengue is transmitted mainly by the bite of* Aedes aegypti *[4]. However,* Aedes albopictus *has also been identified as a secondary vector [5].* A. aegypti *is an urban transmitted mosquito that feeds on multiple hosts during daytime [6]. This behaviour has greatly contributed to the epidemic transmission of dengue [7]. Hence, control of vector is immensely important to reduce the epidemic outbreaks.

Use of insecticide is the major tool for controlling vector-borne diseases including dengue. Organochlorines, organophosphates, pyrethroids, and carbamates are the classes of insecticides that are widely used for vector control [8]. Organochlorines such as dichlorodiphenyltrichloroethane (DDT), and benzene hexachloride (BHC) were widely used in India and Pacific Basin countries for agricultural and public health purposes including controlling malaria for over five decades [9]. In Sri Lanka also DDT was heavily used in controlling malaria by indoor residual spraying mainly during the five-year period, from 1958 to 1963, and was found to be very effective and successful [10]. However, these chemicals were identified as persistent organic pollutants; their adverse effects on the environment and biota causing environmental contamination and effects on wildlife and humans are well documented [9]. Hence, these chlorinated insecticides are banned or severely restricted and alternate insecticides such as organophosphates, carbamates, and pyrethroids were introduced [9]. Pyrethroids are commonly used in mosquito control, wood preservation, impregnation of wool carpets, and textiles [11]. Pyrethroids have been known to enter the aquatic environment from agricultural runoff or drift from aerial or ground based spraying, and these applications may pose threat to fish population by direct exposure, particularly young fish that are less tolerant to these pesticides [11].

Pesguard FG 161™ is a commercial formulation containing d-tetramethrin (4% w/w) and cyphenothrin (12% w/w) which is widely used against dengue vector control [12]. The d-tetramethrin (3,4,5,6-Tetra hydrophthalimidomethyl (1*RS*)-*cis-trans*-chrysanthemate) acts as a knockdown agent and cyphenothrin ((S)-alpha-cyano-3-phenoxybenzyl (1R, 3R)-2,2- dimethyl-3-(2-methylprop-1-enyl) cyclopropanecarboxylate) acts as killing agent; the synergetic activity effectively controls both larval and adult stages of* A. aegypti*,* Aedes albopictus*,* Anopheles sinensis*, and* Culex quinquefasciatus *[12]. During dengue outbreaks, this binary mixture is used as space sprays (either as ground or as air) to achieve rapid control of adult mosquito in the affected area [13]. In some instances, a higher concentration of mixture is used as a larvicide [12]. The space spray application of Pesguard FG 161™ in Sri Lanka has resulted in 100% adulticide activity within 10 m distance [13] and represents the most effective and frequently used strategy for the control of adult mosquito.

D-tetramethrin and cyphenothrin are synthetic pyrethroids [14]. Pyrethroids are chemically synthesized analogues for natural pyrethrin, the extracts of the ornamental* Chrysanthemum cinerariaefolium, *and its related species [15]. Pyrethroids exert excellent insecticidal properties, low mammalian toxicity, and low bioaccumulation [14]. D-tetramethrin is classified as type I pyrethroids without a cyano moiety at the *α*-position, while cyphenothrin belongs to type II pyrethroids that contain an *α*-cyano moiety at the benzylic carbon of the alcohol portion of the ester [16]. Pyrethroids are neurotoxins in general; both types I and II classes prolong the opening of voltage-gated sodium ion channels of the mosquito [17]. In addition, cyphenothrin (type II) affects chloride and calcium channels of nerve filaments by disturbing the GABA (gamma-aminobutyric acid) receptors in the nerve filaments [17].

Although the use of insecticides is recommended to control vector-borne diseases, the risk associated with the overuse should be meticulously investigated. Pyrethroids are normally absent in natural water unaffected by human activities [16]. Insecticides reach surface water from direct disposal, unused residues, indiscriminate application or misapplication, spray drifts, and runoff [16]. Pyrethroids that are used as urban insecticides can reach surface water through concrete drainage systems that are present in suburban and urban areas [18]. Although pyrethroids have apparently shown low toxicity to mammals, they are highly and acutely toxic to fish [19]. The hypersensitivity of fish to pyrethroids is partially explained by their slow metabolic rate, the high sensitivity of the nervous system to pyrethroids and, readily absorption through the gills [16, 20].

Zebrafish model provides valuable insight into environmental risk assessment of chemicals such as pesticides, biocides, and pharmaceuticals [21]. This model has also been recommended as an alternative to fish acute toxicity [22, 23]. Zebrafish, scientifically known as* Danio rerio*, is a small freshwater fish with an approximate length of 2–4 cm. Small size, short life cycle, high fecundity, large brood size, and transparent embryos which develop outside the mother along with rapid development with most organ primordia being formed at 24 hrs after fertilization have made zebrafish ideal subjects in fish embryo toxicity (FET) tests [24, 25]. In zebrafish model of toxicity, a series of adverse morphological phenotypes and behavioural endpoints are used to predict the mechanism of action for toxicity [25].

Although the mammalian toxicology of pyrethroids is well established, relatively few studies have been carried out on fish embryo toxicity. DeMicco et al. (2009) reported that a dose-dependent mortality of zebrafish embryos was observed when exposed to type I pyrethroids (permethrin, resmethrin, and bifenthrin) and type II pyrethroids (deltamethrin, cypermethrin, and l-cyhalothrin) except for resmethrin [22]. However, the effect of d-tetramethrin and cyphenothrin and their synergism on fish embryos has not been investigated. Hence, the present study intended to investigate the acute toxic effect of a binary mixture of d-tetramethrin and cyphenothrin on zebrafish embryos. The finding of this study may broaden the existing knowledge of pyrethroid toxicity. Moreover, our study may encourage the sustainable use of insecticides in controlling vector-borne diseases which subsequently reduce the risks and impacts on the environment, leading to the implementation of Integrated Pest Management (IPM) strategies with alternative approaches or techniques, such as nonchemical alternatives.

## 2. Materials and Methods

### 2.1. Insecticide

A water-based Pesguard FG 161™ containing d-tetramethrin 4 g L^−1^ (12069.64 *μ*molL^−1^) and cyphenothrin l2 g L^−1^ (31960.79 *μ*molL^−1^) formulation recommended for both Ultra Low Volume (ULV) and thermal fogging purposes was obtained from the National Dengue Control Unit, Sri Lanka.

### 2.2. Ethical Consideration of the Study

This study involved the embryo toxicity up to 72 hrs and ethical approval was not required for the study. According to the US National Institutes of Health, zebrafish are considered live animals at hatching, which is approximately 72 h after fertilization [26].

### 2.3. Maintenance of Zebrafish Brood Stock

Brood stock maintenance was carried out according to the OECD guideline no. 236 [27]. Wild-type zebrafish of age between 3 and 6 months were purchased from an aquarium in Colombo and maintained as a brood stock in the laboratory of the Department of Zoology, The Open University of Sri Lanka. Females and males were kept separately in glass aquaria (length 80 cm: height 50 cm: width 46 cm) providing sufficient space for swimming (i.e., ≥1 L per fish). Standardized dilution water (294.0 mgL^−1^ CaCl_2_·2 H_2_O: 123.3 mgL^−1^ MgSO_4_·7 H_2_O: 63.0 mgL^−1^ NaHCO_3_: 5.5 mgL^−1^ KCl) as recommended by ISO 7346-1 and 7346-2 [28, 29] was used for fish maintenance. Fish were maintained at 26±1°C; natural dark/light cycle of 12 hrs was provided with continuous aeration. The water pH was adjusted to 6.8 to 8.4 (using HCl and NaOH). Fish were fed twice a day with artificial diets (Aquaplus, Aqua fish food, India). Frequent removal of food and faeces guaranteed that the ammonia, nitrite, and nitrate are kept below detection limits (0–5, 0.025–1, and 0–140 mgL^−1^, respectively). However, the levels of ammonia, nitrite, and nitrate were measured using commercially available kits (Aquaplus, India) for confirmation.

### 2.4. Spawning of Zebrafish

Eggs were obtained by the random pairwise mating of zebrafish at a ratio of 2:1 of male to female [27]. During the spawning condition females were easily distinguished from males by the swollen bellies. The glass breeding tanks (L 26 cm: H 12.5 cm: W 20 cm) with a green colour wire mesh (1.25 mm) fixed were used for spawning to prevent the eggs from being cannibalized by the adults. Same dilution water was used for spawning. Artificial plants served as breeding stimulant and substrate. Continuous aeration was supplied. Fish were added to the breeding tank immediately before the onset of darkness.

### 2.5. Collection of Eggs

Spawning and fertilization take place within 30 min after the onset of light in the morning. Eggs were collected within one hour of the onset of light. About 30–60 min after spawning, adult fish and wire mesh were removed and the eggs were transferred to a petri dish (60 mm) containing standardized dilution water (50 eggs per each plate) by means of a plastic pipette. Fertilized eggs were selected using an inverted microscope (A.Krüss, Optronic, Germany). Fertilized eggs were identified by presence of cleavage of embryos.

### 2.6. Embryotoxicity Assay

A binary mixture of insecticide was tested for 5 (d-tetramethrin: 0.01 – 1.20 *μ*molL^−1^ and cyphenothrin: 0.02 – 3.20 *μ*molL^−1^) concentrations prepared as dilutions with standard dilution water. Thirty fertilized eggs were randomly selected and transferred into 60 mm petri dish containing different concentrations of binary mixture of d-tetramethrin and cyphenothrin that are used for the assay, the positive control (4 mgL^−1^ (24.69 *μ*molL^−1^) 3,4-dichloroaniline) [27] (Sigma, USA), and negative control (only dilution water) [27], respectively, to minimize the dilution of concentration from the transfer straight from dilution water and to minimize the delay in exposure.

Twenty embryos were individually exposed in 24-well microtiter plates (Corning, USA), each well containing 2 ml of the respective concentrations of a mixture of insecticide. Plates were pretreated with respective solutions to minimize the absorption of the plastic material [27]. A single 24-well plate was used for each concentration of test, negative control, and positive control. Twenty wells of each plate were used for test solution and the remaining 4 wells served as internal control (containing dilution water). The plates were covered with non-adhesive foil and kept at RT (26°C) away from direct sunlight for 24, 48, and 72 hrs [27]. Assays were initiated within 2 hrs of post-fertilization.

### 2.7. Detection of Toxicological Endpoints

At 24, 48, and 72 hour time intervals, embryos of each concentration were observed by an inverted microscope (SZ61, Olympus, Japan). Toxicological endpoints such as coagulation (CA), nondetachment of the tail (NDT), lack of somite formation (LSF), and lack of heartbeat (LHB) [27] were observed under a compound microscope (CH30, Olympus, Japan) and captured using a camera (Tucsen, China). Further, hatching of embryos was also observed at 72 hrs.

### 2.8. Statistical Analysis

Percentage mortalities were calculated for each concentration at different time intervals. Mean values of two independent experiments were obtained. LC_50_ and confidence interval (CI) values were calculated using the probit method in SPSS package [30]. Graphs were plotted using SigmaPlot 14.

## 3. Results

### 3.1. LC_50_ Values of a Binary Mixture of d-Tetramethrin and Cyphenothrin of Zebrafish Embryo Toxicity

**Figure 1:**
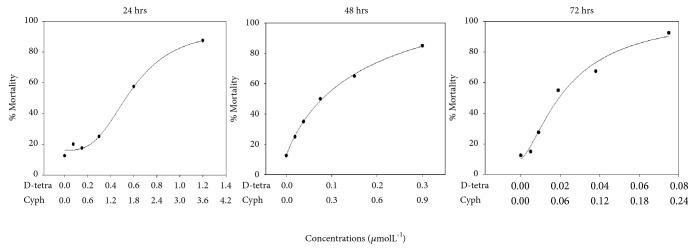
**Graphical representation of % mortality of zebrafish embryos against different concentrations at 24, 48, and 72 hour time intervals. **The curves were fitted into 4-parameteric sigmoidal hill curve using SigmaPlot 14 software. Mean values of percentage mortality of two different experiments were plotted against concentrations. 0% is minimum value and 100% is maximum value.

The exposure of Zebrafish embryos (within 2 hours after fertilization) to different concentrations of a binary mixture of tetramethrin and cyphenothrin revealed that the toxicity is time and concentration dependent. As indicated in Table 1, the LC_50_ values at 24 hrs (d-tet: 0.58 *μ*molL^−1^, cyp: 1.74 *μ*molL^−1^) were significantly reduced in 48 hrs (d-tet: 0.11 *μ*molL^−1^, cyp: 0.33 *μ*molL^−1^) and 72 hrs (d-tet: 0.03 *μ*molL^−1^, cyp:0.09 *μ*molL^−1^). The LC_50_ value at 24 hrs was reduced nearly by 20 times at 72 hrs. The LC_50_ values exhibited significant negative correlation with the time of exposure (P<0.05). The chi-square values for different time intervals were not significant and thus indicate that embryo populations are not homogenous. The dose response curves of the mixture for 24, 48, and 72 hrs are represented in Figure 1. The curves were fitted into 4-parameter sigmoidal hill curves.

The embryo mortality was found to be less than 12.5 % in control experiments at different time intervals. Further, positive control (4 mgL^−1^ (24.69 *μ*molL^−1^) 3, 4-dichloroaniline) exhibited more than 80% of embryo mortality even at 24 hrs of exposure. There was no embryo mortality in the internal control. Low mortality in the negative control and higher mortality in the positive control validate the reliability of the experiment.

Batches of twenty fish embryos were exposed to five different concentrations of a binary mixture of d-tetramethrin and cyphenothrin (diluted in dilution water). Mortality was recorded every 24-hour interval up to 72 hrs in two independent experiments and mean values of percentage mortality were calculated. LCL and UCL denote the lower and upper confidence limits, respectively, for the LC_50_ values.

### 3.2. Types of Lethal Endpoints Observed during the Exposure

**Figure 2:**
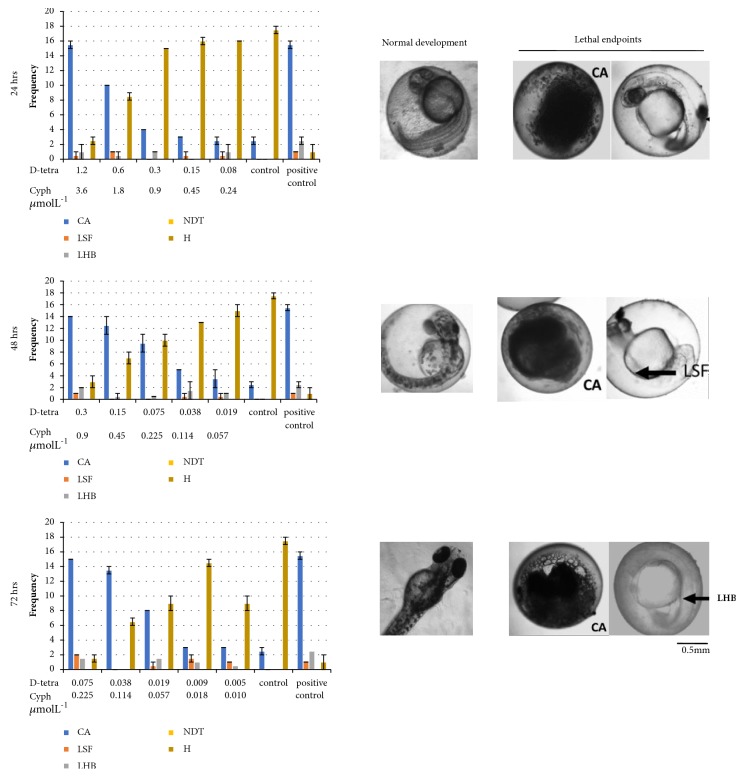
**Types and frequencies of lethal effects observed in zebrafish embryos when exposed to binary mixture of d-tetramethrin and cyphenothrin at different time intervals: 24, 48, and 72 hrs.** CA: coagulation, LSF: lack of somite formation, LHB: lack of heartbeat, NDT: None-detachment of tail, H: number of hatched embryos D-tetra: tetramethrin, Cyph: Cyphenothrin. Control: dilution water, positive control (4 mgL-1 (24.69 *μ*molL-1) 3,4-dichloroaniline).

The data on lethal effects at 24- to 72-hour exposure are summarized in Figure 2. The observation of results indicates that coagulation (Figure 2) is the most common form of lethal effect that was observed. Lack of somite formation and lack of heartbeat were also visible in some embryos as represented in Figure 2. The numbers of zebrafish hatched at each time interval are also presented in Figure 2. The embryo that did not have any lethal effects was hatched after 72 hrs of the experiment.

The embryos that exhibited the lethal effects also showed low pigmentations and late development. However, the hatched larvae did not show any sublethal effects after 72 hrs when the experiment was stopped due to ethical considerations.

## 4. Discussion

Pesguard FG 161™ which consists of d-tetramethrin and cyphenothrin (1:3) is extensively used for dengue vector control and the environmental impact is less studied. In environmental toxicological studies, fish toxicity holds a greater promise. Most of the fish toxicity studies have been carried out using adult or juvenile fish [3, 31]; however, ethical concerns have been raised against using live fish [32]. Zebrafish embryo toxicity model has been proposed as the most promising alternative approach to classical acute fish toxicity testing with live fish [32]. Higher fecundity and rapid development have allowed testing a number of samples at shorter duration [33]. Moreover, the transparent nature of embryos makes them easy to visualize morphological and developmental abnormalities during testing [33]. Egg development outside the mother provides the ability to expose the eggs directly to the toxicants as well as allowing dose determination in comparison to mammals which require admission of the toxicant to the mother [22]. Further, zebrafish model has been recommended as a suitable model to evaluate chiral pesticides [33].

Our results revealed that the Pesguard FG 161™ binary mixture of d-tetramethrin and cyphenothrin is highly toxic to zebrafish embryo. The toxicity increases with the time of exposure and test embryos were observed at 72 hrs. The apparent difference in LC_50_ values could be attributed to the different stages of zebrafish and synergistic effect of the two pyrethroids. Moreover, the commercial formula of Pesguard FG 161™ may contain other substances that may also contribute to the toxic effect. Hence, in the future this study will be repeated using the pure form of pyrethroids.

It is argued that the lethal endpoints of zebrafish embryo toxicity may not accurately represent sensitive neurotoxic effects and juvenile or adult may be more sensitive to pyrethroids [33]. Moreover, it is suggested that the stereochemistry of pyrethroids can also influence the toxicity. Generally, the cis isomer of pyrethroids is more toxic than the trans isomer for mammals [34] with a sensitivity of fish to stereochemistry depending on the pyrethroid type used [16]. However, Kent (1996) demonstrated that d,d,trans-cyphenothrin is not expected to differ significantly from that of cyphenothrin [35]. However, the LC_50_ values obtained for binary mixture at 48 and 72 hrs in our experiment were lower than those in the previous experiment indicating a synergetic effect of both d-tetramethrin and cyphenothrin. Further, other environmental factors (temperature, water, pH, total hardness, and dissolved oxygen) can also affect the toxicological assays.

Tetramethrin was found to be highly toxic to freshwater Rainbow trout (*Oncorhynchus mykiss*) with an LC_50_ value of 3.7 *μ*g/L (0.01 *μ*molL^−1^) (flow through) and 21 *μ*g/L (0.06 *μ*molL^−1^) (static) for 96 hrs, respectively [36]. Similarly, cyphenothrin exhibited an LC_50_ value of 0.38 *μ*g/l (0.001 *μ*molL^−1^) at 96 hrs for Rainbow trout in the acute flow-through system [36]. Both tetramethrin and cyphenothrin were found to be highly toxic to Rainbow trout compared to that of Zebrafish embryo. The Rainbow trout is also a valuable model for studying toxic effect of chemicals [37]. The high sensitively of Rainbow trout to these pyrethroids may partially attribute to the notion that the zebrafish embryo may not well reflect the lethality caused by neurotoxic chemicals. The neurotoxins may affect the locomotion of fish which could subsequently affect the oxygen intake from gills, leading to death [38]. These effects are absent in embryos as the oxygen intake mainly occurs through diffusion [38]. Therefore, embryos could be less sensitive to pyrethroids than adult or juveniles of fish. Even though the model is less sensitive to pyrethroids considering the ethical concern using large number of fish this model can be used as an effective alternative method to assess toxicity of fish.

One of the biggest challenges in zebrafish embryo toxicity assay is the difficulty in maintaining concentrations and often the actual exposure concentrations can be significantly lower than the concentration used in the test [38]. Hence, the reduction in the sensitivity of the zebrafish embryo could be attributed to the difference in the test and exposure concentration. Thus, further experiments are warranted to confirm the exposure concentration of the assay. Furthermore, the sensitivity of zebrafish embryos to the pyrethroid can be validated by conducting a parallel study using juvenile fish.

It is reported that pyrethroids are up to 1000 times more toxic to fish than mammals at comparable equivalent concentrations [16]. The sensitivity of fish to pyrethroids, compared with other vertebrates, is mainly explained by the slow rate of biotransformation [16]. Fish are deficient in the pyrethroid hydrolyzing enzymes [39]. In addition, the sensitivity of a fish nervous system to pyrethroids and readily absorption through the gills to the bloodstream of the fish make them more vulnerable to pyrethroid toxicity [16, 20].

Coagulation of eggs was the most common lethal effect observed in all concentrations and time of exposure of the binary mixture of pyrethroids followed by lack of somite formation and lack of heartbeat. The previous experiment conducted on malathion also reported coagulation of embryo as the common lethal effect [40] and it is consistent with our observation. All viable embryos observed in each concentration were hatched at 72 hrs. Though the present study concentrated on lethal effects, identification of sublethal and teratogenic deformation is essential for comprehensive understating of toxicity of these pyrethroids. Hence, further studies are encouraged.

In the current test, the individual eggs were obtained from two different brood stocks. It is believed that the replicates spread among different batches of eggs produce more reliable results. Low mortality levels in control (12.5%) and higher mortality in the positive control (over 80%) indicate the reliability of the experiment. Further, none of the embryos died in internal control wells; hence it is assumed there was no effect from the inert material from the plate.

Although pyrethroids pose low toxicity to mammals, transient dizziness, headache, nausea, anorexia, and fatigue symptoms could be manifested in humans from mild acute occupational exposure [41]. Dermal exposure to pyrethroids may cause temporary skin irritation and paresthesia localized to the exposure site.

The application of pyrethroids outdoors and in water bodies is restricted due to high toxicity to fish and other aquatic organisms [42]. Instead, they are applied as ultralow volume (ULV) application (also known as thermal or cold fogs) [43]. The occurrence of two massive eel (*Anguilla anguilla*) devastations in Lake Balaton, Hungary, in the years 1991 and 1995 found deltamethrin, the active ingredient of the antimosquito insecticide K-OTHRIN 1 ULV, to be the causative agent. Bream (*Abramis brama*), Pike perch (*Stizostedion lucioperca*), and Common Gull (*Larus canus*) were several other animal species found affected [44]. Although space spraying can rapidly reduce adult mosquito populations, regular reapplication is necessary to maintain control [42]. Thus, during outbreaks, a significant amount of insecticides is released to the environment. The insecticides in spray forms may drift with the wind and rain and may wash into surface water. In addition, disposal of residuals, and inappropriate usage such as washing the spray equipment, can contaminate the water bodies [45].

Consistent with our study, the previous experiment also shows that pyrethroids are detrimental to fish and other aquatic organisms even at the acute exposure [46]. Hence, contamination of water bodies with these insecticides should be minimized. Generally, pyrethroids persist only for a short time and are rapidly degraded by sunlight (photodegradation), chemical reactions in water (hydrolysis), and the action of microbes (biodegradation) [47]. Therefore, more studies should be conducted to evaluate the residual levels of these chemicals in water bodies during dengue outbreak seasons.

Although the insecticides have saved millions of lives from malaria and plague, their long-term impact on the environment could be devastating [48, 49]. Hence the competing public concern on controlling vector-borne disease and environmental risk associated with the use of insecticides should be balanced.

## 5. Conclusion

The experimental data observed in this study reveals the health risk of Pesguard FG 161™ d-tetramethrin and cyphenothrin in combination with non-target organisms such as fish. The binary mixture of pyrethroids exhibited lethal endpoints in zebrafish embryos; hence this model can be used as an alternative model to protected stages of fish. As embryos are less sensitive to neurotoxins such as pyrethroids, further studies are recommended. The ultimate objective of this study is to reduce the disease burden in a sustainable and equitable manner. These threats to the ecosystem could easily become a reality if pyrethroids are not well maintained.

## Figures and Tables

**Table 1:** LC_50_ values of a binary mixture of d-tetramethrin and cyphenothrin at different time intervals.

**Exposure time (hrs) **	**L** **C**_50_ ** (** ***μ*** **mol** **L** ^−1^ **) ** **d- tetramethrin: cyphenothrin**	**95**%** Confidence limits of LC**_50_** (*μ*molL**^−1^** ) **		**r** ^2^	**Chi-square **
		**LCL**	**UCL**		

**24 **	0.58:1.74	0.45:1.35	0.77: 2.31	0.98	0.75

**48 **	0.11: 0.33	0.08: 0.24	0.16: 0.48	0.90	2.79

**72 **	0.03: 0.09	0.02: 0.06	0.04: 0.12	0.93	3.57

## Data Availability

The [mortality] data used to support the findings of this study are included within the article
